# Toward a New Paradigm for Targeted Natriuretic Peptide Enhancement in Heart Failure

**DOI:** 10.3389/fphys.2021.650124

**Published:** 2021-10-13

**Authors:** Olof Gidlöf

**Affiliations:** Department of Cardiology, Clinical Sciences, Lund University, Lund, Sweden

**Keywords:** heart failure, natriuretic peptide system, ARNI, epigenetics, RNAi, gene therapy, non-coding RNAs

## Abstract

The natriuretic peptide system (NPS) plays a fundamental role in maintaining cardiorenal homeostasis, and its potent filling pressure-regulated diuretic and vasodilatory effects constitute a beneficial compensatory mechanism in heart failure (HF). Leveraging the NPS for therapeutic benefit in HF has been the subject of intense investigation during the last three decades and has ultimately reached widespread clinical use in the form of angiotensin receptor-neprilysin inhibition (ARNi). NPS enhancement via ARNi confers beneficial effects on mortality and hospitalization in HF, but inhibition of neprilysin leads to the accumulation of a number of other vasoactive peptides in the circulation, often resulting in hypotension and raising potential concerns over long-term adverse effects. Moreover, ARNi is less effective in the large group of HF patients with preserved ejection fraction. Alternative approaches for therapeutic augmentation of the NPS with increased specificity and efficacy are therefore warranted, and are now becoming feasible particularly with recent development of RNA therapeutics. In this review, the current state-of-the-art in terms of experimental and clinical strategies for NPS augmentation and their implementation will be reviewed and discussed.

## Introduction

Heart failure (HF) is a serious and complex clinical condition characterized by inadequate ventricular filling or ejection of blood (Yancy et al., 2013). It arises as the result of structural or functional impairments of the heart, caused by a wide range of underlying diseases, including e.g., hypertension, ischemic heart disease and valvular disease (Ziaeian and Fonarow, 2016). An estimated 37.7 million people suffer from HF (Vos et al., 2012), and the number of patients is rapidly rising as a result of shifts in global age distribution. Symptoms of HF include fatigue, dyspnea, poor exercise tolerance, and fluid retention and often lead to impaired quality of life and frequent hospitalizations. An important clinical distinction is made between HF patients with reduced and preserved left ventricular ejection fraction (LVEF). The two groups are approximately equal in size, but with significant differences with regards to risk factors, pathophysiology, prognosis, and response to treatment (Ponikowski et al., 2016). HF with reduced (<40%) ejection fraction (HFrEF) is characterized by a weakened myocardium, typically with a dilated left ventricle, resulting in inefficient pumping during systole. HF with preserved (>50%) ejection fraction (HFpEF) is associated with a stiff myocardium with left ventricular hypertrophy and increased filling pressures which result in insufficient relaxation and filling of the heart during diastole.

Standard medical treatment for HFrEF is aimed at reducing cardiac workload and increase diuresis through inhibition of the renin-angiotensin-aldosterone (RAAS) and sympathetic nervous system (SNS), and can confer substantial improvements with regards to mortality and quality of life (Garg and Yusuf, 1995; Packer et al., 1996). Still, morbidity remains high for many patients and the 5-year survival rate after hospitalization is comparable to that of many cancers (Murphy et al., 2020). Moreover, in the case of HFpEF there are currently no evidence-based treatments available. Thus, there is an urgent need for new and improved therapeutic strategies for both HFpEF and HFrEF.

The natriuretic peptide system (NPS)constitutes a beneficial compensatory mechanism in all forms of HF, effectively reducing cardiac pre- and afterload in response to increased cardiac filling pressures by promoting natriuresis, diuresis, and vasodilation (Goetze et al., 2020). Thus, harnessing the NPS for therapeutic benefit has long been considered an attractive strategy and, as a result of extensive research over the last three decades, has recently reached widespread clinical use. The success of angiotensin receptor/neprilysin inhibition (ARNi) in treating HFrEF (McMurray et al., 2014) has been attributed to increased NP activity (Packer et al., 2015; Ibrahim et al., 2019). However, its use is complicated by the fact that neprilysin degrades a number of other vasoactive peptides in the circulation, e.g., glucagon, substance P, and bradykinin, which can cause hypotension and angioedema (McMurray et al., 2014) and raises concerns about long-term adverse effects through accumulation of potentially pathogenic peptides (e.g., amyloid-β) (Galo et al., 2020). Moreover, ARNi has proven ineffective in patients with HFpEF (Solomon et al., 2019). Therefore, alternative approaches to enhance the NPS with increased specificity and potency could lead the way toward more refined and efficacious HF therapies. The aim of this review is to provide an overview of the current state-of-the art in terms of experimental, preclinical and clinical strategies to enhance the NPS for therapeutic benefit in HF.

## The Natriuretic Peptide System

The NPS is a fundamental homeostatic mechanism regulating blood pressure and extracellular fluid volume. Atrial and b-type natriuretic peptides (ANP and BNP) are hormones produced and released from cardiomyocytes in response to mechanical stimuli and stimulates diuresis, natriuresis, and vasodilation (Goetze et al., 2020). Urodilatin is an ANP isoform with four additional N-terminal residues produced primarily in the kidney (Schulz-Knappe et al., 1988). Within the cardiovascular system, C-type natriuretic peptide (CNP) is mainly synthesized by the endothelium, where it is released in response to various stimuli (e.g., increased shear stress and cytokine signaling) and exerts vasodilatory and anti-proliferative effects locally within the vasculature (Fu et al., 2018). Whereas ANP and BNP are intimately associated with HF pathogenesis and progression, CNP is only modestly increased in HF (Wei et al., 1993a; Cargill et al., 1994; Del Ry et al., 2005) and mechanistically not a direct therapeutic target in HF. Therefore, CNP will not be discussed in detail in this review.

### Synthesis and Secretion of Natriuretic Peptides

Atrial and b-type natriuretic peptides are transcribed from the *NPPA* and *NPPB* genes, respectively, which are located within approximately 10 kb of each other on chromosome 1p36.22. The locus is subject to highly coordinated and rigorous spatiotemporal control during development and disease, which is exerted both by epigenetic regulators (Rubattu et al., 2020) and transcription factors (Man et al., 2018). In healthy adults, *NPPA* expression is restricted to atrial cardiomyocytes whereas cardiac *NPPB* expression is generally low (Litvinukova et al., 2020; Tucker et al., 2020). However, the locus is subject to extensive changes in the local chromatin environment (Hohl et al., 2013; Sergeeva et al., 2016) resulting in a coordinated upregulation of both genes in atrial as well as ventricular tissue upon mechanical and neurohormonal stimulation (Saito et al., 1989; Feldman et al., 1991; Sergeeva et al., 2014). Numerous hallmarks of HF, including hemodynamic overload and increased wall stretch, as well as increased neurohormonal signaling through angiotensin II (Lako-Futo et al., 2003; Majalahti et al., 2007), endothelin (Archer et al., 2017), and alpha adrenergic (Knowlton et al., 1991) stimuli all result in potent transcriptional activation of the *NPPA*/*NPPB* locus in both atrial and ventricular tissue. HF-related activation of *NPPA*/*NPPB* gene expression has been demonstrated to be driven partly through mitogen-activated protein kinases (MAPKs), specifically mediated via extracellular signal-related kinase- (ERK-) signaling (Bueno et al., 2000; Kehat et al., 2011; Koivisto et al., 2011), with GATA4(Liang et al., 2001; Kerkela et al., 2002; Tenhunen et al., 2004; van Berlo et al., 2011), NFAT (Molkentin et al., 1998), Myocardin (Kuwahara et al., 2010) being some examples of key transcription factors involved.

Translation of *NPPA* and NPPB mRNA results in 151- and 134-amino acid preprohormones, respectively, which are processed into proANP and proBNP by enzymatic removal of their respective signal peptides (Fu et al., 2018). Biologically active BNP is produced intracellularly by the subtilisin-like proprotein convertase Furin (Nishikimi et al., 2015), whereas proANP is cleaved to form biologically active ANP after secretion by the membrane-bound serine protease Corin (Yan et al., 2000). Under physiological conditions, ProANP and processed BNP are stored together in specific atrial granules and released together in response to mechanical (Mangat and de Bold, 1993) and hormonal stimuli (Ogawa et al., 1999). In contrast, sustained pressure overload and wall stress leads to production and release of these NPs from both atrial and ventricular tissue, reflected in the marked elevation of both ANP and BNP in the circulation of HF patients.

### Natriuretic Peptide Signaling

The natriuretic system includes three known receptors: NPR-A, -B, and -C. The biological functions of ANP and BNP are mediated by binding to natriuretic peptide receptor A (NPR-A), whereas CNP binds primarily to NPR-B. NPR-A and -B are membrane bound guanylate cyclases that, upon binding of their respective ligands produce cyclic guanosine monophosphate (cGMP) which acts as an intercellular second messenger activating protein kinase PKG and phosphodiesterase (PDE) to regulate numerous pathways, including ion channels, protein phosphorylation, nuclear translocation and gene expression (Schlossmann et al., 2005). It has been reported that ANP is 10-fold more potent in stimulating NPR-A than BNP (Koller and Goeddel, 1992). NPR-A expression is particularly high in the arterial system, kidney, adipose tissue, adrenal gland, and lung (Sarzani et al., 1996; Nagase et al., 1997; Mele et al., 2015). In the kidney, NPR-A activation leads to increased glomerular filtration rate (Marin-Grez et al., 1986), inhibition of sodium and water reabsorption (Kishimoto et al., 1996) and reduced secretion of renin (Kurtz et al., 1986). In the arterial system, NPR-A mediates vasorelaxation by decreasing intracellular calcium levels and calcium sensitivity in vascular smooth muscle cells through PKG-I (Carvajal et al., 2000). In the adrenal gland, ANP/NPR-A inhibits adrenocorticotropin- and angiotensin-induced synthesis of aldosterone (Chartier et al., 1984; Kudo and Baird, 1984). Together, these physiological effects mediated by NPs constitute a key homeostatic counterweight to dysregulated SNS- and RAAS-signaling in HF. Interestingly, at concentrations observed in mild HF, ANP is more potent than BNP in inhibiting the aldosterone response to Angiotensin II (Hunt et al., 1996). Additionally, the natriuretic peptide system exerts direct effects in the heart, inhibiting cardiac remodeling and fibrosis. ANP and BNP have been shown to inhibit angiotensin II- and norepinephrine-induced proliferation of cardiac fibroblasts (Fujisaki et al., 1995; Calderone et al., 1998). Genetic approaches aimed at reducing or abrogating ANP/NPR-A signaling receptor in mice leads to a blood pressure-independent exacerbation of cardiac hypertrophy, fibrosis, and left ventricular dysfunction in animal models of HF (Kishimoto et al., 2001; Knowles et al., 2001; Kuhn et al., 2002; Holtwick et al., 2003; Wang et al., 2014). Moreover, carriers of genetic variants in the *NPPA* promoter associated with decreased circulating proANP levels showed increased left hypertrophy (Rubattu et al., 2006).

### Natriuretic Peptide Clearance and Metabolism

Once released from their cell of origin, NPs are rapidly cleared from the circulation. ANP has an estimated half-life of ∼ 2 min in healthy human subjects (Nakao et al., 1986; Yandle et al., 1986), whereas the half-life of BNP is slightly longer, ∼20 min (Mukoyama et al., 1991; Holmes et al., 1993). There are two recognized mechanisms for NP clearance: receptor-mediated degradation and enzymatic proteolysis. The NPR-C receptor, which is bound by all NPs (Suga et al., 1992) and has a similar tissue expression profile as NPR-A (Sarzani et al., 1996), lacks the intercellular GC domain (Sudoh et al., 1990) and is believed to function mainly as a clearance receptor, internalizing natriuretic peptides for lysosomal degradation (Nussenzveig et al., 1990; Cohen et al., 1996). Enzymatic degradation of circulating NPs is mainly carried out by Neprilysin (NEP), a membrane-bound metalloprotease expressed in a wide variety of tissues and cells but is particularly abundant in the kidney (Pavo et al., 2020). Besides NPs, NEP degrades numerous additional vasodilatory peptides including bradykinin (Gafford et al., 1983), substance P (Skidgel et al., 1984), adrenomedullin (Lisy et al., 1998), as well as vasoconstrictors such as angiotensins (Gafford et al., 1983) and endothelins (Skolovsky et al., 1990). NPs are also cleaved by insulin-degrading enzyme (IDE) (Muller et al., 1991, 1992), another zinc-dependent metalloprotease with a wide repertoire of target peptides (Tundo et al., 2017). It should be noted however that BNP is a poor substrate for both NEP and IDE (Potter, 2011), and additional proteases are likely involved in its degradation. A number of animal studies have assessed the relative contribution of Natriuretic peptide receptor C (NPRC)- and NEP-mediated degradation of ANP. Results consistently show that the combination of NPRC-blocking peptides and NEP inhibitors (NEPi) cause a greater increase in circulating ANP (and its downstream physiological effects) than either of the individual treatments alone (Koepke et al., 1989; Kukkonen et al., 1992; Okolicany et al., 1992; Charles et al., 1996). Although these results suggest that both pathways are equally important in degrading ANP, it has been postulated that NEP plays a predominant role in the clearance of patho-physiological levels of ANP, when clearance receptors are believed to be saturated (Hashimoto et al., 1994).

## Current Therapies Targeting the Natriuretic Peptide System

The concept of utilizing NPs for clinical benefit in HF dates back to the 1980s, after the potent natriuretic and vasodilatory properties of ANP was discovered (de Bold et al., 1981; Kangawa and Matsuo, 1984). To date, two principal approaches for therapeutic augmentation of the NP system have been utilized: (1) administration of synthetic NPs and (2) inhibition of NP degradation.

### Synthetic Natriuretic Peptides

Findings that infusion of human alpha-atrial natriuretic peptide led to increased natriuresis and arterial pressure in healthy subjects (Richards et al., 1985) and reduced pulmonary arterial wedge pressure, systemic vascular resistance and increased stroke volume in patients with congestive HF (Crozier et al., 1986; Riegger et al., 1986; Saito et al., 1987; Goy et al., 1988) spurred the development of synthetic NPs for clinical use. Intravenous injection of Carperitide, recombinant ANP, was approved for the treatment of acute decompensated HF in Japan in 1995, but evidence for its long-term benefit on cardiac function, clinical symptoms or prognosis have not been confirmed in large-scale, randomized clinical trials, and the drug has not reached widespread clinical use. Ularitide, a chemically synthesized analogue of the urodilatin, showed short-term beneficial hemodynamic effects in patients with congestive and decompensated HF with both reduced and preserved EF in early clinical trials (Elsner et al., 1995; Mitrovic et al., 2005, 2006) but failed to reduce long-term cardiovascular mortality in the larger, double-blind TRUE-AHF trial, including patients with acute HF (of which 35% had preserved LVEF) (Packer et al., 2017). Nesiritide, a 32 amino acid form of recombinant BNP effectively reduced cardiac pre- and afterload in patients with congestive HFrEF (Mills et al., 1999) and was subsequently approved for the treatment of acute decompensated HF in the United States in 2001. However, since the large-scale, randomized clinical ASCEND-HF trial failed to show any benefit on mortality or re-hospitalization of Nesiritide compared to placebo in acute HF, it is no longer recommended for routine use (O’Connor et al., 2011).

Taken together, the results of numerous clinical trials (summarized in Table 1) over the last three decades shows that while administration of exogenous, synthetic NPs can provide short-term effects on hemodynamics and natriuresis in patients with acute HF, their use is limited by lack of long-term effects on mortality or hospitalization, a requirement for intravenous administration, and side effects such as severe hypotension.

**TABLE 1:** Clinical trials—synthetic NPs in HF.

**Study**	**Synthetic NP**	**Patient group (n)**	**Main outcomes**
Crozier et al., 1986	ANP	CHF (7)	PCWP/RAP ↓
			CO ↑
Saito et al., 1987	ANP	CHF (6)	PCWP ↓
			SV ↑
			Diuresis/Natriuresis ↑
Goy et al., 1988	ANP	HFrEF (8)	PCWP ↓
			CI ↑
Elsner et al., 1995	Urodilatin	HFrEF (12)	SBP/CVP ↓
			Diuresis/Natriuresis ↑
Mitrovic et al., 2005	Urodilatin	DHF (24)	PCWP ↓
			RAP ↓
Mitrovic et al., 2006	Urodilatin	DHF (221)	SVR ↓
			CI ↑
Packer et al., 2017	Urodilatin	AHF (2157, 477 HFpEF)	SBP ↓
			Cardiovascular death—NS
			Hospitalization—NS
Mills et al., 1999	BNP	HFrEF (103)	PCWP ↓
			RAP ↓
			SVR ↓
			CI ↑
			SV ↑
O’Connor et al., 2011	BNP	AHF (7141, 1048 HFpEF)	Death or rehospitaliztion for HF—NS

*CHF, congestive heart failure; HFrEF, heart failure with reduced ejection fraction; DHF, decompensated heart failure; AHF, acute heart failure; HFpEF, heart failure with preserved ejection fraction; PCWP, pulmonary capillary wedge pressure; RAP, right atrial pressure, CO, cardiac output; SV, stroke volume; CI, cardiac index; SBP, systolic blood pressure; CVP, central venous pressure; SVR, systemic vascular resistance; NS, not significant.*

### Inhibition of Natriuretic Peptide Degradation

While infusion of exogenous NPs has thus far been ineffective in treating HF, increasing the amount of biologically active NPs in the circulation through NEPi has eventually proven to be a more successful therapeutic strategy (Table 2). The first proof-of-concept was established in 1989, when intravenous infusion of thiorpan, a selective NEPi, resulted in increased plasma ANP, increased natriuresis/diuresis and reduced pulmonary arterial wedge pressure and atrial pressure in patients with mild chronic HFrEF (Northridge et al., 1989). Beneficial effects was later seen with another intravenously administered NEPi, candoxatril, in patients with more severe HFrEF (Munzel et al., 1992), but the compound failed to reduce blood pressure and systemic vascular and pulmonary resistance in subsequent clinical trials and development was discontinued (McDowell and Nicholls, 1999). Similarly, a dose-ranging trial with the oral NEPi ecadotril failed to show significant effects on neuroendocrine measures and symptoms in chronic HFrEF patients (Cleland and Swedberg, 1998). These initial failures were attributed to the fact that NEPi-mediated NP augmentation was counteracted by the parallel increase in other vasoactive peptides degraded by NEP, in particular vasoconstrictors such as Angiotensin I, II, and Endothelin-1 (Gafford et al., 1983; Skolovsky et al., 1990). In line with these observations, it was also noted that the effect of angiotensin converting enzyme inhibitors (ACEi) on exercise capacity in patients with mild chronic HFrEF was improved with the addition of candoxatril (Newby et al., 1998). These insights led to the development of the orally active, combined ACEi-NEPi compound omapatrilat (Robl et al., 1997). Although proving to be more potent in reducing blood pressure and improving hemodynamics than candoxatril (McClean et al., 2000; Rouleau et al., 2000), omapatrilat failed to show meaningful benefit with regards to mortality and hospitalization compared to ACEi alone in the large, randomized double-blind OVERTURE trial, which included >5,700 HFrEF patients (Packer et al., 2002). In addition, omapatrilat was shown to be associated with increased occurrence and severity of angioedema compared to ACEi alone in the OCTAVE trial (Kostis et al., 2004), which ultimately meant the drug never reached clinical use. The increased risk of angioedema was attributed to elevated levels of bradykinin, a result of the simultaneous inhibition of three bradykinin-degrading proteases: ACE, NEP, and aminopeptidase (Sulpizio et al., 2005), resulting in excessive vasodilation and vascular permeability. This problem was circumvented by instead combining NEPi with angiotensin receptor blockade (ARB), which was shown to provide NP augmentation and inhibition of angiotensin signaling without disrupting ACE-mediated bradykinin degradation (Hegde et al., 2011). The orally active, first-in-class angiotensin receptor NEP inhibitor (ARNi) LCZ696 (sacubitril-valsartan) was safe and well tolerated and associated with increased plasma NP levels, increased diuresis and lowered blood pressure in phase I and II studies (Gu et al., 2010; Solomon et al., 2012). A phase III trial also showed that LCZ696 reduced diastolic and systolic blood pressure compared to valsartan alone in patients with mild to moderate hypertension. Importantly, LCZ696 was well tolerated and not associated with increased risk of angioedema (Ruilope et al., 2010). The PARADIGM-HF phase III, randomized, double-blind trial included >8,000 HFrEF patients was designed to compare the effects of LCZ696 and the ACEi enalapril on a composite end point of death from cardiovascular causes or hospitalization for HF. The trial was terminated early because the pre-specified limit for an overwhelming benefit of LCZ696 had been reached. Compared with enalapril, LCZ696 reduced death from any cardiovascular cause by 20% and hospitalization from HF by 21%. In secondary analyses, LCZ696 was also shown to provide reduced risk of non-fatal clinical deterioration compared to enalapril (Packer et al., 2015). Patients in the LCZ696 group were less likely to require intensified HF treatment or to be hospitalized for worsening HF compared to patients in the enalapril group. There was also a trend toward patients on LCZ696 being less likely to require mechanical assist device implantation or cardiac transplantation. On the molecular level, a marked and sustained decrease of plasma NT-proBNP and troponin, biomarkers of cardiac wall stress and injury respectively, was observed in the LCCZ696 group. Interestingly, a separate study found that LCZ696 also reduced markers of fibrosis (sST2, TIMP-1, Gal-3, PNP, and PIINP) and collagen degradation (MMP-2 and -9) compared to enalapril, providing indirect evidence that NEPi-mediated NP-augmentation also benefits the myocardium, which may contribute to improved clinical outcomes (Zile et al., 2019). Indeed, compared to enalapril, LCZ696 was recently shown to reduce the risk of sudden cardiac death (Shen et al., 2017), a complication of HF to which fibrosis is a known risk factor (Gulati et al., 2013). More evidence for a beneficial effect of ARNi on the myocardium was published in two separate studies recently, showing that LCZ696 improved indices of ventricular volume and function (left ventricular end-diastolic and -systolic volume index, left atrial volume index and early diastolic annular velocity) compared to enalapril both short-(Desai et al., 2019) and long term (Januzzi et al., 2019).

**TABLE 2:** Clinical trials—inhibition of NP degradation in HF.

**Study**	**Drug**	**Mode of action**	**Patient group (n)**	**Main outcomes**
Northridge et al., 1989	Thiorpan	NEPi	HFrEF (6)	Diuresis/Natriuresis ↑
				PCWP ↓
Munzel et al., 1992	Candoxatril	NEPi	HFrEF (9)	CI ↑
				Diuresis/Natriuresis ↑
				PCWP ↓
Cleland and Swedberg, 1998	Ecadotril	NEPi	HFrEF (279)	Plasma/urinary cGMP ↑
				Symptoms/QoL scores—NS
Packer et al., 2002	Omapatrilat	ACEi-NEPi	HFrEF (5770)	Death/Hospitalization in HF—NS
McMurray et al., 2014	Sacubitril-Valsartan	ARNi	HFrEF (8442)	Death/Hospitalization in HF ↓
Solomon et al., 2019	Sacubitril-Valsartan	ARNi	HFpEF (4822)	Death/Hospitalization in HF—NS
Zile et al., 2019	Sacubitril-Valsartan	ARNi	HFrEF (2067)	Profibrotic biomarkers ↓
Desai et al., 2019	Sacubitril-Valsartan	ARNi	HFrEF (464)	LAVI ↓
				LVEDVI ↓
				LVESVI ↓
				E/e’ ↓
Januzzi et al., 2019	Sacubitril-Valsartan	ARNi	HFrEF (794)	NT-proBNP ↑
				LVEF ↑
				LVEDVI ↓
				LVESVI ↓
				LAVI ↓
				E/e’ ↓

*NEPi, neprilysin inhibition; ACEi; angiotensin converting enzyme inhibition; ARNi, angiotensin receptor neprilysin inhibition; HFrEF, heart failure with reduced ejection fraction; HFpEF, heart failure with preserved ejection fraction; PCWP, pulmonary capillary wedge pressure; CI, cardiac index; QoL, quality of life; NS, not significant; LAVI, left atrial volume index; LVEDVI, left ventricular end-diastolic volume index; LVESVI, left ventricular end-systolic volume index; LVEF; left ventricular ejection fraction.*

The favorable effects of ARNi have been attributed to increased NP availability and signaling, but the contribution of each individual NP family member has not been elucidated. Differences in the affinity of NEP for the individual NPs (CNP ≥ ANP >> BNP)(Kenny et al., 1993; Watanabe et al., 1997) suggests that NEPi would affect plasma levels of CNP and ANP to a greater extent than BNP. Nevertheless, the investigators of the PARADIGM-HF trial associated the beneficial effects of LCZ969 with increased levels of BNP, based on a relatively modest (16%) increase in the levels of the prohormone NT-proBNP (McMurray et al., 2014). However, the substantially larger (63%) increase in plasma cGMP suggested that other NPs contributed to the overall effect of ARNi. Ibrahim et al. (2019) recently carried out a comprehensive analysis of plasma NPs and a range of NP cleavage products over time in HFrEF patients on LCZ696 using a wide variety of assays. While the difference in BNP was inconsistent and showed considerable inter-assay variability and the levels of NT-proBNP actually decreased, ANP was elevated in a rapid, potent and sustained manner in response to ARNi. CNP levels were generally low and did not appear to be affected by the treatment. These results points toward ANP as an important mediator of the beneficial effects of ARNi.

Despite the undoubted success of ARNi in the treatment of HF, there are a number of limitations and precautions to consider. First, while LCZ696 has proven effective in reducing mortality and hospitalization in HFrEF, ARNi appears to be less effective in HFpEF. The PARAGON-HF trial assessed the effect of LCZ696 compared to valsartan alone in HF patients (NYHA class II–IV) with EF >45% (Solomon et al., 2019). After a median of 35 months follow-up, no significant differences were observed with regards to the primary endpoint (a composite of hospitalization for HF and cardiovascular death) between the treatment groups. In secondary analyses, LCZ696 showed significant benefit in women and in patients with mid-range EF (46–57%), suggesting that ARNi might be effective in certain subgroups of HFpEF patients. Second, there is a concern regarding the numerous other vasoactive peptides targeted by Neprilysin, and the potential side effects that ARNi might cause. Although safety and tolerability of NEPi therapy was considerably improved by the combination with ARB, the occurrence of angioedema and hypotension is still higher with ARNi compared to ACEi (McMurray et al., 2014; Solomon et al., 2019). Moreover, serious concerns have been raised regarding the risk of NEPi and Alzheimer’s disease (AD). Amyloid-β is a well-established NEP substrate (Shirotani et al., 2001; Kanemitsu et al., 2003) and NEPi in animals results in a substantial increase in amyloid-β and the appearance of plaque-like deposits in the brain, similar to those associated with AD in humans (Iwata et al., 2000). Although no increase in incidence of AD or neurological adverse effects were observed during the 4.3 year follow-up of PARADIGM-HF (McMurray et al., 2014) or the 36-week time course of the PARAMOUNT-HF trial (Solomon et al., 2012), the development of AD occurs over a considerably longer period of time. A multi-center, randomized, double-blind trial (PERSPECTIVE, ClinicalTrials.gov identifier: NCT02884206) to comprehensively evaluate the longitudinal effects of LCZ696 on cognitive function (including memory, executive function, and attention) in patients with HFpEF is currently ongoing and will hopefully be able to address these concerns.

## Novel Approaches for Therapeutic Natriuretic Peptide Augmentation

**FIGURE 1:**
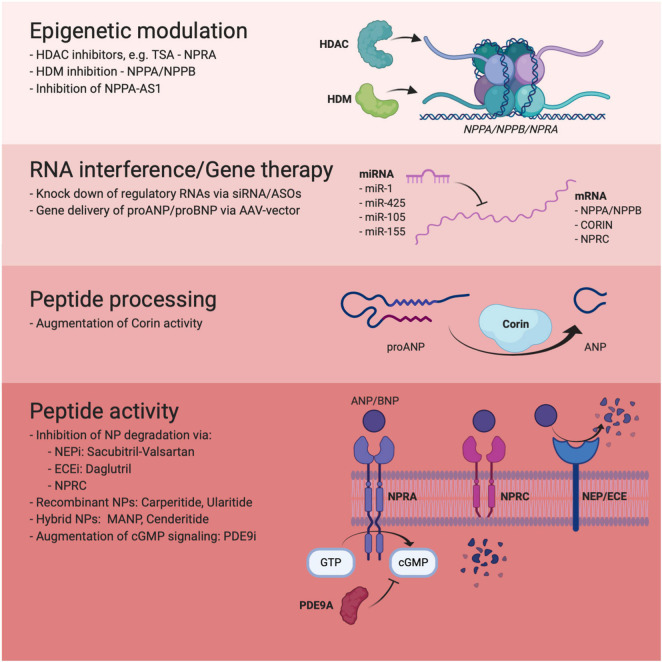
Overview of novel approaches to therapeutic NP augmentation included in this review.

As discussed above, ARNi represents a shining example and proof-of-concept of how harnessing the NP system can be used for therapeutic benefit in HF. However, the ubiquitous and promiscuous nature of Neprilysin makes it less than ideal as a drug target and the potential long-term side effects of ARNi have yet to be fully characterized. Currently, a wide range of alternative therapeutic strategies, involving regulation of NP synthesis, processing and signaling are being explored in experimental, preclinical and clinical studies with the aim to provide more specific and efficacious NP augmentation therapy (summarized in Figure 1).

### Epigenetic Approaches to Natriuretic Peptide Augmentation

Epigenetic drugs, i.e., drugs modulating the state of chromatin, via targeting e.g., DNA methyltransferases, histone deacetylases (HDACs) and Bromodomain and Extra-terminal motif (BET) proteins have reached widespread clinical use in cancer and chronic inflammatory diseases (Nebbioso et al., 2018; Nemtsova et al., 2019). The use of epigenetic drugs have also shown promising effects in experimental models of HF. For example, both the BET inhibitor JQ1 and the broad-spectrum HDAC inhibitor trichostatin A have been shown to suppress pathological cardiac hypertrophy and fibrosis in pressure-overload models of HF (Kong et al., 2006; Anand et al., 2013; Duan et al., 2017). The genetic locus encompassing *NPPA* and *NPPB* is subject to extensive epigenetic regulation in response to mechanical and neurohormonal stimuli, both via changes in DNA methylation and through modifications of chromatin, allowing a more permissive transcriptional environment. In experimental models of phenylephrine- and myocardial infarction-induced cardiac hypertrophy and failure, activation of the *Nppa* promoter was shown to be mediated by the histone acetyltransferase (HAT)-activity of p300 (Gusterson et al., 2003; Miyamoto et al., 2006). A marked increase in H3K9-acetylation was also observed in the promoter of the *Nppa* and *Nppb* genes in response to pressure overload in mice (Sayed et al., 2013). Moreover, the histone demethylase JMJD2A was shown to mediate angiotensin II and endothelin-1- induced increases in *NPPB* expression in human induced pluripotent stem (iPS) cell derived cardiomyocytes (Rosales and Lizcano, 2018) and its expression was also found to be upregulated in human failing myocardium, accompanied by a decrease in the repressive chromatin modification H3K9me2 and H3K9me3 in the *NPPA* and *NPPB* promoters of human failing hearts (Hohl et al., 2013). An epigenome-wide analysis of cardiac tissue from HF patients with dilated cardiomyopathy (DCM) identified significant hypomethylation across the *NPPA*/*NPPB* locus, indicative of transcriptional activation (Meder et al., 2017). NP receptors are also subject to epigenetic control. Overexpression of HDAC1 and HDAC2 significantly enhanced *Npr1* promoter activity and expression of *Npr1* in primary mouse mesangial cells (Kumar et al., 2014b). Moreover, HDAC inhibition promoted recruitment of HATs to the *Npr1* promoter and significantly elevated renal *Npr1* expression, GC activity and cGMP levels *in vivo* (Kumar et al., 2014a). These studies show that epigenetic mechanisms actively regulate the NP system at multiple levels, that many of these mechanisms are active in the failing myocardium and that they could potentially be exploited for therapeutic NP augmentation. However, pharmacological tools for precise tuning of the chromatin environment within the heart are not yet available (Napoli et al., 2020).

### RNA-Based Therapeutic Targets Within the Natriuretic Peptide System

The concept of RNA as therapeutic targets in cardiovascular medicine is gaining increasing traction, with RNA-based drugs for hypercholesterolemia (Inclisiran) and cardiac amyloidosis (Patisiran) being FDA- and EMA-approved or in late stage clinical trials (Leiter et al., 2019; Minamisawa et al., 2019).

The advantages of RNA as drug targets include ease of design and production as well as the ability to target any gene with high specificity and the potential to affect targets that are “undruggable” on the protein level. In theory, once a disease-associated target transcript has been identified, a chemically modified, complementary antisense oligonucleotide (ASO) or small interfering RNA (siRNA) can be synthesized and, once delivered to the cell or tissue of interest, modulate the expression, translation or splicing of the target (Levin, 2019). By leveraging genomic information, therapeutic antisense oligonucleotides with high specificity for the target transcript can be produced, minimizing the risk for off-target effects. One class of transcripts of particular interest as therapeutic targets are non-coding, regulatory RNAs (Gomes et al., 2020). This class encompasses both short (microRNAs) and long (long non-coding RNAs, lncRNAs) which regulate gene expression. MicroRNAs bind to complementary sequences, primarily in the 3’ untranslated region (UTR), of mRNAs to repress translation or mediate mRNA degradation (Bartel, 2009), whereas lncRNAs regulate gene expression through a wide range of mechanisms, including antisense binding, transcription factor recruitment and modulation of the local chromatin environment (Palazzo and Koonin, 2020). Tissue- and context-specific expression and a high degree of conservation make regulatory RNAs attractive as potential therapeutic targets. Across the various stages of NP synthesis, processing, and signaling, there are a number of potential RNA targets that could be harnessed for therapeutic benefit in HF. The first approach that will be covered here is targeting regulatory RNAs with negative effects on NP synthesis and processing. The NP genes are themselves subject to such regulation by both short and long regulatory RNAs. Arora et al. (2013) were the first to describe a regulatory RNA involved in NP synthesis. A genetic variant associated with blood pressure, situated in the 3’UTR of *NPPA*, was found to alter the binding and regulatory capacity of microRNA-425 (miR-425). Thus, miR-425 silenced *NPPA* expression in an allele-specific manner and was proposed as a potential target for specific upregulation of ANP. Subsequently, through a comprehensive transcriptomic and genetic screening and various *in vitro* assays performed by the same constellation of researchers, miR-105 and miR-155 were also shown to regulate *NPPA* expression in a similar manner (Wu et al., 2016; Vandenwijngaert et al., 2018). Interestingly, a recent clinical trial found the expression of miR-425 in atrial tissue to be significantly elevated in black individuals compared to white participants (Patel et al., 2019), an observation which might in part explain what has been touted as an NP-deficient state of black individuals (Gupta et al., 2015a,b, 2017; Bajaj et al., 2018). Overlapping the *NPPA* gene is a natural antisense transcript, *NPPA-AS1*, with potential regulatory capacity (Halley et al., 2013). Our research group showed that *NPPA-AS1* expression is localized to cardiomyocyte nuclei, is responsive to mechanical stimuli and is elevated in the myocardium of HF patients. Mechanistically, *NPPA-AS1* was shown to negatively regulate *NPPA* expression through interaction and recruitment of the repressive transcription factor RE1-silencing transcription factor (REST) to the *NPPA* promoter. *In vivo* inhibition of mouse Nppa-as1 resulted in increased cardiac and circulating Anp, reduced blood pressure and increased renal cGMP signaling (Celik et al., 2019b). In our view, this makes *NPPA-AS1* an interesting target for NP augmentation and work is currently ongoing to elucidate the therapeutic benefit of *Nppa-as1* knock down in models of HFpEF and HFrEF.

While no evidence has been published showing direct regulation of *NPPB* by non-coding RNAs, there are a number of studies showing how miRNAs negatively affect processing of proBNP. Two separate studies have demonstrated that *FURIN* expression is regulated by miR-24, but neither investigated the potential downstream effects on BNP synthesis and signaling (Luna et al., 2011; Wang et al., 2012). Nakagawa et al. (2017) found that miR-30 regulates the expression of GalNAc-transferases 1 and 2, and thereby the extent to which proBNP is glycosylated at threonines 48 and 71, and as a result, the amount of secreted proBNP. With regards to processing of proANP, our group recently performed a functional screening to identify microRNA regulators of Corin activity in human iPS-derived cardiomyocytes. miR-1, a cardiac-enriched microRNA, was identified as a particularly potent inhibitor of Corin activity through direct binding to a target site in *CORIN* mRNA. Interestingly, miR-1 was also found to have multiple additional targets involved in the transcription and processing of ANP (Celik et al., 2019a).

An alternative approach for RNA-based NP augmentation is targeting NP clearance receptor expression and/or function. Two microRNAs that regulate NPR-C expression have been discovered thus far. Using a combination of bioinformatic screening and transcriptome analysis, Wong et al. (2015) identified miR-100 as a potential regulator of *NPR3* expression and subsequently validated a direct miRNA:mRNA interaction *in vitro*. Later, Wang et al. (2018) described a mechanism whereby miR-143 exerts repressive effects on *NPR3* expression in cardiomyocytes. Of note, the authors also showed that the levels of miR-143 were elevated in the circulation of HF patients. Based on these results, both miR-100 and miR-143 could constitute potential RNA-based targets to achieve an increased level of circulating NPs. A more direct approach was taken by Venkatesan et al. (2016), who used an siRNA-based strategy to directly target the *Npr3* gene. In an isoproterenol-induced model of HF, intramyocardial injection of *Npr3* siRNA resulted in increased circulating levels of Anp and reduced cardiac hypertrophy and fibrosis. While promising, these findings should be taken with caution as NPR-C has been shown to play roles beyond NP clearance (Rubattu et al., 2010), for example with regards to bone growth (Matsukawa et al., 1999) and cardiac conductance (Rahmutula et al., 2019).

Although potent *in vivo* modulation of gene expression can be achieved relatively easily through administration of ASOs or siRNAs, there are a number of considerations and concerns that must be taken into account. For example, delivery of RNA-based drugs to organs other than the liver has proved to be challenging. Conjugation of ASOs or siRNAs with peptides (Ammala et al., 2018), sugar derivatives (Akinc et al., 2010), or aptamers (Catuogno et al., 2019) can provide tissue-specific targeting in some contexts, but an effective route for myocardial delivery has yet to be discovered.

Another issue, which is particularly relevant with regards to the targeting of regulatory RNAs, is specificity. As discussed above, microRNAs are inherently pleiotropic, with the potential of binding a wide repertoire of mRNA targets. The effects of long regulatory RNAs are more heterogenous by nature, exerting influence over a number of genes in a specific locus through modulation of the chromatin conformation (Khalil et al., 2009), or acting on a single target gene through antisense binding (Faghihi et al., 2008). Thus, careful evaluation of transcriptome-wide effects of therapies based on regulatory RNAs is warranted. Based on the studies included here (summarized in Table 3) numerous interesting targets for RNA-based augmentation of the NP system exist, but important challenges remain before they can reach clinical use.

**TABLE 3:** Putative targets for RNA-based augmentation of the NP system.

**Target**	**Type of target**	**Effect**	**References**
miR-425	microRNA	Repression of *NPPA*	Arora et al., 2013
miR-105	microRNA	Repression of *NPPA*	Wu et al., 2016
miR-155	microRNA	Repression of *NPPA*	Vandenwijngaert et al., 2018
miR-1	microRNA	Repression of *CORIN*	Celik et al., 2019a
miR-24	microRNA	Repression of *FURIN*	Luna et al., 2011; Wang et al., 2012
miR-30	microRNA	Regulates proBNP glycosylation/secretion	Nakagawa et al., 2017
miR-100	microRNA	Repression of *NPR3*	Wong et al., 2015
miR-143	microRNA	Repression of *NPR3*	Wang et al., 2018
NPPA-AS1	lncRNA	Repression of *NPPA*	Celik et al., 2019b
NPR3	mRNA	siRNA-mediated knock down of *NPR3*	Venkatesan et al., 2016

### Modulation of Enzymatic Activity in Natriuretic Peptide Synthesis and Processing

Inhibition of the metalloprotease Neprilysin is the obvious example of how altering enzymatic activity can be utilized to enhance the NP system, but other enzymes within the NP pathway have also been touted as potential therapeutic targets. There is evidence to suggest that the expression and activity of Corin is decreased in the development and progression of HF, (Chen et al., 2010; Dong et al., 2010; Ibebuogu et al., 2011; Tripathi et al., 2016) resulting in dysregulated proANP processing and less biologically active ANP in the circulation. Restoring Corin activity can therefore be viewed as a potential therapeutic strategy in HF. Experimental support for this approach was provided in a study by Gladysheva et al. (2013), where transgenic mice overexpressing Corin showed reduced pulmonary congestion as well as improved systolic function and survival compared to wild type mice in a DCM-like model of HF. However, it is important to acknowledge that enhancement of Corin activity could lead to accumulation of other, yet unidentified target peptides, and with that, potentially undesired effects.

Endothelin-converting enzyme (ECE) is a transmembrane metalloprotease primarily known to produce biologically active Endothelin-1 (ET-1) from its precursor peptide PPET1. ET-1 is a highly potent vasoconstrictor which counteracts the beneficial effects of the NP system in HF (Giannessi et al., 2001). ECE shares structural features with Neprilysin and was shown both in experimental (Johnson et al., 1999) and physiological (Nakayama et al., 2012) settings to also actively degrade ANP. Thus, inhibition of ECE (ECEi) could provide beneficial neurohormonal outcomes by simultaneously decreasing ET-1 and increasing ANP. Combined ECEi and NEPi was subsequently shown to result in sustained improvement in systolic function and reduced cardiac remodeling compared to NEPi alone in ischemic and hypertensive models of HF (Mulder et al., 2004; Emoto et al., 2005; Kalk et al., 2011). In clinical trials, administration of the oral ECEi-NEPi Daglutril in healthy human subjects resulted in a significant increase in preproET-1 and ANP (Seed et al., 2012) and reduced pulmonary capillary wedge pressure and atrial pressure in patients with HFrEF (Dickstein et al., 2004). While these studies have established proof-of-concept, the long-term effects of ECE-NEPi on survival and hospitalization in HF have not been investigated.

### Natriuretic Peptide Gene Therapy

With the advent of recombinant adeno associated viral (AAV) vectors, gene therapy is now a clinical reality for the treatment of diseases such as hemophilia and spinal muscular atrophy (High and Roncarolo, 2019). Cardiomyocytes, being terminally differentiated, non-dividing cells, should in theory constitute promising target cells for AAV-vectors, and the idea of using gene therapy to elevate cardiac NP expression has been explored in a number of experimental studies during the last 25 years. In pioneering work, Lin et al. (1995) showed that injection of naked plasmid DNA encoding ANP caused a potent and sustained lowering of blood pressure in young (but not adult) spontaneously hypertensive rats (SHR). Later, the same research group showed that adenoviral delivery of human ANP increased diuresis and natriuresis, lowered blood pressure and attenuated cardiac hypertrophy in Dahl salt-sensitive rats on a high-salt diet (Lin et al., 1998). More recently, Cataliotti et al. (2011) designed a myocardium-tropic AAV serotype 9 vector encoding preproBNP that, upon injection in SHR produced cardiomyocyte-specific overexpression of BNP and elevated plasma BNP as well as a sustained reduction in systemic blood pressure and improved diastolic and systolic function. In a later study, injection of an AAV9-vector encoding rat proBNP inhibited worsening of cardiac function and significantly prolonged survival in SHR (Tonne et al., 2014). Although these animal studies have shown promising results, targeting the heart with gene therapy has thus far been a challenge in humans. The experience from the CUPID and CUPID2b trials, where the effect of AAV1-mediated delivery of sarcoplasmic/endoplasmic reticulum Ca^2+^ ATPase 2a (SERCA2a) on hospitalization for or ambulatory treatment for worsening HFrEF, showed that while safe, the treatment did not confer significant clinical benefit (Jaski et al., 2009; Jessup et al., 2011; Zsebo et al., 2014; Greenberg et al., 2016). The lack of effect has been attributed to inefficient delivery of the vector to cardiac cells, with an estimated <2% of cardiomyocytes containing a vector in the group receiving the highest dose. Thus, increasing transduction efficiency must be improved before myocardial gene therapy becomes clinically useful as a means of increasing cardiac NP production.

### Designer NPs

The limited clinical success of infusion of recombinant, native NP (discussed above) has prompted the development of engineered hybrid NPs to improve pharmacological profiles and minimize undesirable effects. Vasonatrin (VNP) is a synthetic peptide consisting of the full-length 22-amino acid CNP and the 5-AA C-terminus of ANP (Wei et al., 1993b). It has been shown to stimulate both NPR-A and -B (Jiang et al., 2014) and to be a more potent vasorelaxant than ANP (Wei et al., 1993b). In an animal model of ischemic cardiomyopathy, infusion of VNP was shown to improve hemodynamic parameters through a cGMP/PKG-dependent mechanism (Shi et al., 2015).

Cenderitide, a chimeric NP consisting of the full-length 22 AA human CNP and the 15-AA C-terminus of DNP, an NP isolated from *dendroaspis*, was designed in order to reduce the risk of systemic hypotension, a common and serious side effect of recombinant ANP and BNP, while retaining potent renal effects (Lisy et al., 2008). Infusion of Cenderitide was shown to be safe and to increase plasma cGMP and urinary sodium excretion in healthy subjects (Lee et al., 2009) and in a recent randomized clinical trial, increased plasma cGMP and urinary cGMP excretion without affecting blood pressure in patients with HFrEF (Kawakami et al., 2018).

Mutant ANP (mANP) is the result of a familial frame-shift mutation in exon 3 of *NPPA*, which gives rise to an ANP isoform with 12 additional C-terminal amino acids (Hodgson-Zingman et al., 2008). mANP is more resistant to NEP-mediated degradation (Dickey et al., 2009) and produces more potent natriuretic, diuretic and hemodynamic effects than ANP when administered *in vivo* (McKie et al., 2009). In a canine model of acute Ang II-induced hypertension with elevated cardiac filling pressures, infusion of mANP caused a significantly lowered pulmonary wedge pressure, artery pressure and right atrial pressure, increased urinary cGMP and reduced aldosterone levels as compared to infusion with human BNP (McKie et al., 2010). Importantly, the increased stability of mANP makes it suitable for subcutaneous administration (Chen et al., 2020), and does not require intravenous infusion like native recombinant NPs. In a randomized, double-blind, placebo-controlled trial, subcutaneous administration of mANP (ZD100) resulted in a sustained decrease in blood pressure and a reduction in aldosterone levels in patients with resistant hypertension, a major driver of HF (Chen et al., 2016). An additional phase I clinical trial to evaluate the cardiovascular properties of mANP administration in African Americans with resistant hypertension is currently recruiting (ClinicalTrials.gov identifier: NCT04542681), but the long-term effects of mANP on blood pressure as well as potential outcomes in patients with established HF has yet to be elucidated.

### Modulation of Downstream Signaling

Augmentation of GC-A/cGMP/PKG signaling downstream from the NPR-A receptor could represent another possible approach for utilizing the NP system therapeutically in HF. Stimulation of the related guanylate cyclase sGC, the downstream effector of nitric oxide (NO)-signaling, via oral administration of Vericiguat was recently shown to reduce death and hospitalization in patients with HFrEF (Armstrong et al., 2020). While activation of the NO/cGMP pathway can be achieved with NO-independent sGC-stimulators, similar pharmacologic tools are currently unavailable for stimulating GC-A. Phosphodiesterases (PDEs) negatively regulate the cGMP signal by hydrolyzing cGMP to GMP, and could be considered therapeutic targets with relevance to the NP pathway. Lee et al. (2015) revealed that PDE9A is upregulated in the failing human heart and inhibits NP- rather than NO-dependent cGMP signaling. Inhibition of PDE9A was subsequently shown to protect the myocardium from neurohormonal and hemodynamic stress. Interestingly, oral PDE9A inhibitors are available and well tolerated in humans (Schwam et al., 2014; Moschetti et al., 2016; Brown et al., 2019) and might constitute a future approach to HF treatment.

## Summary and Future Perspectives

Heart Failure is a disease characterized by neurohormonal imbalance, and the NP system has been recognized as a beneficial equipoise to RAAS- and SNS-activation and a potential therapeutic target for three decades. Finding the right approach to enhance the NP system has however been a long and arduous endeavor, and even though the success of ARNi means that it has finally found its way to widespread clinical use (in HFrEF), alternative routes for more refined, precise and potent NP augmentation should still be explored. As evident from the many experimental and clinical studies presented here, the NP system can be targeted on multiple levels with diverse therapeutic modalities. Importantly, each approach also comes with specific challenges. A common hurdle for epigenetic, RNAi- and gene therapy-based therapies is the difficulties of cardiac drug delivery. The identification of cardiomyocyte-specific antigens or receptors to allow development of antibody-, peptide- or oligomer-conjugated drug delivery is a crucial step in taking these therapies toward clinical use. Increasing our understanding of the transcriptome and chromatin-state of single cardiac cells and cell-types will also help in designing epigenetic and RNA-based drugs with greater specificity and less risk of off-target effects. Improvements in vector design, delivery methods and dosage have the potential to enhance efficacy of cardiac gene therapy. With regards to enhancing NP half-life and activity beyond NEPi, it is interesting to note that the primary enzyme responsible for BNP degradation is likely yet to be discovered. Identification of this enzyme can thus lead to even more potent NP augmentation. Enhancing NP-signalling downstream of the NPR-A receptor is also an interesting therapeutic prospect for future studies. Pharmacological inhibition of the cardiac-specific cyclic phosphodiesterase PDE9A could represent a potent NP-like stimulus to treat HF-induced hypertrophy and fibrosis.

The distinct lack of clinical trials focusing on the large group of HF patients with preserved ejection fraction (HFpEF) is worth mentioning. The failure of the PARAGON-HF trial to show benefit of ARNi in patients with HFpEF is disappointing, but not entirely unsurprising given the relative heterogeneity of this patient group. Targeted approaches such as those described above would likely have a better chance of providing benefit, especially if combined with precision medicine approaches (Gori et al., 2020).

To conclude, a broad spectrum of possible therapeutic targets exists within the NP system beyond Neprilysin, all with the promise of improving HF treatment and all associated with specific challenges that must be resolved prior to clinical implementation. The research field is ripe with experimental and clinical studies addressed to meet these challenges and ultimately provide the next generation of NP augmentation therapy.

## Author Contributions

The author conceived the idea, performed literature search, and wrote the manuscript.

## Conflict of Interest

The author declares that the research was conducted in the absence of any commercial or financial relationships that could be construed as a potential conflict of interest.

## Publisher’s Note

All claims expressed in this article are solely those of the authors and do not necessarily represent those of their affiliated organizations, or those of the publisher, the editors and the reviewers. Any product that may be evaluated in this article, or claim that may be made by its manufacturer, is not guaranteed or endorsed by the publisher.
